# An Accurate Anchor-Free Contextual Received Signal Strength Approach Localization in a Wireless Sensor Network

**DOI:** 10.3390/s24041210

**Published:** 2024-02-14

**Authors:** Nour Zaarour, Nadir Hakem, Nahi Kandil

**Affiliations:** Telebec Underground Communications Research Laboratory, University of Quebec at Abitibi-Temiscamingue, 675, 1e Avenue, Val-d’Or, QC J9P 1Y3, Canada; nadir.hakem@uqat.ca (N.H.); nahi.kandil@uqat.ca (N.K.)

**Keywords:** wireless sensor network, network connectivity context, RSS, inter-node estimation, anchor-free, small/large-scale fading channel, localization

## Abstract

Sensor localization remains a crucial function within the context of wireless sensor networks (WSNs) and is a delicate concern that has attracted many researchers’ attention. Undoubtedly, a good distance estimation between different wireless sensors allows us to estimate their accurate locations in the network well. In this article, we present a simple but very effective anchor-free localization scheme for wireless sensor networks called the contextual received signal strength approach (CRSSA) localization scheme. We use the received signal strength (RSS) values and the contextual network connectivity within an anchor-free WSN. We present and thoroughly analyze a novel joint estimation methodology for determining the range, path loss exponent (PLE), and inter-node distances in a composite fading model that addresses small-scale multipath fading and large-scale path loss shadowing effects. We formulate analytical expressions for key parameters, the node’s communication range and the PLE value, as functions of the sensor’s number, the network’s connectivity, and the network density. Once these parameters are estimated, we estimate the inter-node distances and the positions of nodes, with relatively high accuracy, based on the assumed propagation model in a two-dimensional anchor-free WSN. The effectiveness of the CRSSA is evaluated through extensive simulations assuring its estimation accuracy in anchor-free localization.

## 1. Introduction

Wireless sensor networks (WSNs) are a fundamental component in a wide range of applications, including environmental monitoring, object tracking, logistics management, and industrial operations [1]. These networks consist of small, cost-effective sensor nodes that communicate wirelessly, and the precise localization of these nodes within the network is essential for their effective operation. These networks, comprising small, cost-effective sensor nodes, may require precise localization for effective operation. A review published in [2] exposed location-aware schemes, as well as parameters affecting the performance of localization during the past few years. Within the realm of WSN localization, techniques are broadly categorized into two main types: anchor-based and anchor-free methods. Anchor-based methods rely on nodes with known positions to assist in the localization process, offering high accuracy but often at the cost of reduced flexibility and increased operational expenses. In contrast, anchor-free methods, which do not depend on predefined anchor positions, offer greater flexibility and scalability, making them increasingly popular in dynamic and large-scale deployment scenarios [3,4,5].

Traditionally, localization has often relied on anchor nodes with known positions. Although these algorithms have high localization accuracy, anchors introduce limitations in deployment flexibility and incur higher operational costs. Consequently, there is a growing interest in developing and refining anchor-free algorithms, which promise a flexible structure and reduced cost. Our research directly addresses the challenges and opportunities in anchor-free localization within WSNs [6,7]. In our endeavor, we introduce the “contextual received signal strength approach” (CRSSA), an innovative method leveraging the widely utilized received signal strength (RSS) metric, compatible with a broad range of commercially available sensors. This universality is a key strength, enabling diverse sensors to efficiently process RSS data extracted from standard received communication packets. Although RSS-based localization is celebrated for its simplicity and widespread applicability, it grapples with accuracy challenges stemming from the complexities and unpredictability of signal propagation, especially in industrial environments. Our previous work in developing CRSSA for one-dimensional (1D) environments showed promising results [8]; however, the problem was confined to a basic linear context or string topology. In this article, the primary research question that we address is whether extending CRSSA to two-dimensional (2D) WSNs maintains its precision and avoids divergence. Moving beyond the linear limitations of the 1D context, we aim to explore the capabilities of CRSSA in the more complex and spatially diverse 2D environments, ensuring that the transition to a higher dimensional space does not compromise the method’s accuracy or lead to algorithmic instability. By thoroughly investigating signal behavior complexities, including multipath effects and environmental interference, we strive to affirm the robustness and reliability of the CRSSA for diverse and demanding conditions in industrial WSN applications [6,7].

Our research encompasses a comprehensive simulation evaluation, designed to rigorously evaluate the CRSSA in a 2D setting. We compare the performance of the 2D-extended CRSSA against established anchor-free algorithms, with a focus on determining whether the shift to 2D preserves the accuracy and stability observed in 1D environments. Special emphasis is placed on evaluating the precision of inter-node distance estimation, a critical factor in assessing the effectiveness of any localization method. The forthcoming sections will thoroughly explore the theoretical underpinnings of the 2D CRSSA, delve into the intricate signal propagation challenges in 2D environments, and present detailed results from simulations. These insights will culminate in a discussion on the practical implications of the findings, particularly how the 2D CRSSA could be implemented in real-world wireless sensor networks, addressing the unique challenges that they present.

## 2. Related Works

### 2.1. Anchor-Free Localization

In the field of anchor-free localization within WSNs, several techniques and algorithms have been proposed. Youssef et al. [9] introduced a clustering-based approach, dividing the network into clusters, each with its gateway node responsible for generating a local relative map. These local maps are then combined to obtain the global network layout. However, it is worth noting that managing these clusters can introduce significant complexity into the localization process. Arbula et al. [10] also employed clustering but in a distributed manner, initially localizing clusters and then integrating them together to achieve network-wide localization. While cluster-based approaches offer advantages in certain scenarios, they frequently require intricate coordination and management, adding to the overall complexity of the localization system. The work in [11] took advantage of the sensor clustering approach to organize the network topology and the triangle test method to determine cluster heads. A local coordinate system was formed within each cluster, and a fusion algorithm was proposed to unify all clusters into a uniform coordinate system. In the work referenced as [12], Wang et al. introduced a distributed cluster-based anchor-free node localization algorithm by employing a clustering approach for single-hop nodes and the time of arrival (ToA) technique to estimate inter-node distances. Then, a cluster synchronization was employed, where all nodes within a cluster are harmonized, and local coordinates are determined based on angle and distance information. The final step was the global localization phase. The most recent work in [13] presented an efficient anchor-free algorithm (EAFLA), which grouped nodes into one-hop clusters and estimated distances between nodes within each cluster using RSS. For nodes not within one hop, the Kleincrock model [14] and Al-Kashi’s theorem (cosine law) were used to derive the positions of sensors in the network. In contrast, our CRSSA streamlines the localization process by avoiding the complexities associated with clustering. Utilizing contextual connectivity information alongside the readily available RSS metric, the CRSSA aims to achieve precise localization without the overhead of managing multiple clusters or gateway nodes, thereby simplifying operations and reducing costs.

The authors in [15] proposed a range-based anchor-free approach, where distances between all pairs of sensors within communication range were known, while distances beyond this range were considered unknown. Contrasting with this approach, the CRSSA operates on the principle that no distances are pre-known, even among proximate sensors. This challenges the traditional reliance on pre-established distances within communication range, as seen in [15]. An anchor-free localization approach was introduced in [16], utilizing a single node as a global reference position. This node sends a special active packet through the network, and when received by a sensor, the sensor would calculate the angle and distance to the node. However, this approach required all sensors to be equipped with angle-measuring devices. The authors in [6] proposed the ladder diffusion node localization algorithm (LDLA) anchor-free localization algorithm, in which each sensor calculated its relative position with the sink node. Activation packets were broadcast by the sink node, and when received, a sensor would relay them using the ladder diffusion method. This allowed sensors to adjust their coordinate systems based on received packets from source nodes and neighbor node positions, but this method also shared the same limitation of requiring angle-measuring devices for effective localization. The work in [7] presented a method that achieved positioning through beacon data interaction between a rotating antenna on the base station and sensor nodes. The sensors determined their positions based on the vertical elevation angle of the antenna, the horizontal rotation angle, and distance information from the node to the base station antenna. In comparison, the CRSSA approach diverges significantly by being designed to work with the inherent capabilities of almost all commercial sensors, eliminating the need for specialized hardware. This adaptability makes the CRSSA not only more cost-effective but also more versatile, as it can be implemented in a wider range of environments without the need for complex setups or additional equipment.

In [17], the authors utilized the LDLA approach and introduced the sunflower algorithm (SFO) for implementing range-free anchor-free localization in WSNs using distance vector-hop (Dv-Hop) and considering the distance vector routing protocol. This method adopted a radio channel model based on free space attenuation, a simplification that may not align with the complexities of real-world signal propagation in various environments. While this approach provides a basic framework, it potentially overlooks the nuanced signal behaviors often encountered in environments with physical obstructions or interference. CRSSA diverges from this conventional one, adopting a more realistic view of signal propagation behavior in WSN environments. CRSSA enhances the accuracy and reliability of localization, adapting to the diverse and dynamic nature of signal interactions. This adaptability is particularly crucial in real-world applications where signal propagation can be unpredictable and complex. Table 1 summarizes anchor-free localization studies as well as used techniques and approaches.

In localization techniques utilizing RSS, it is essential to consider the precision and variability of the signal across different fading models. However, the majority of localization studies in WSN assume a propagation model where the received power is related to distance by a path loss model with zero-mean Gaussian noise, such as in [18,19,20]. An anchor free algorithm for one-hop nodes is proposed in [21], where it computes the inter-node distances based on RSS and the centroid techniques. The nodes are then located by using the average of the inter-node distances. However, this algorithm does not provide highly accurate position estimates, especially in situations with a high degree of measurement noise or non-line-of-sight conditions, and it does not take into account signal propagation characteristics. An anchor-based hybrid localization algorithm in the absence of knowledge of the transmit power and PLE value is presented in [18]; these parameters are estimated using a Kalman filter, and an unknown node is localized based on RSS/AOA (angle of arrival) information. The authors in [19] proposed a method that utilized the RSS value at the unknown node and the most valuable player algorithm to localize it in the grid system. Also, in [20], the authors proposed anchor-based range-free algorithms based on RSS measurements, namely support vector regression (SVR) and SVR + Kalman filter (KF). On the other hand, many studies, including [22,23], emphasize the importance of accurate signal propagation models in wireless communication systems, especially when accounting for phenomena like multi-path fading and shadowing effects. Traditional models like the lognormal distribution for large-scale shadowing effects [24] and the gamma distribution as an alternative [25] have their limitations. In realistic environments, where these phenomena coexist, a composite fading distribution becomes necessary. For our CRSSA method, we have utilized the Nakagami-m model, as detailed in [26,27,28]. This model is particularly effective in capturing the variability of signal fading in diverse environmental conditions. Employing the Nakagami-m model enhances the adaptability and accuracy of the CRSSA, ensuring its effectiveness across a range of WSN scenarios and solidifying it as a robust solution for the complexities of real-world signal propagation.

**Table 1:** Anchor-free localization approaches.

Issue/Paper	Technique and Approach
Youssef et al., 2005 [9]	In order to obtain the global network layout, the authors proposed an anchor-free clustering-based approach to generate local maps that are then combined together.
Arbula et al., 2008 [10]	The authors employed clustering but in a distributed manner, initially localizing clusters and then integrating them together to achieve network-wide localization.
Xingfu et al., 2010 [11]	The authors used a clustering technique with the triangle test method to form a local coordinate system.
Qu et al., 2015 [16]	The authors introduced an anchor-free localization approach, where the sensor would calculate the angle and distance to a single node as a global reference position.
Wang et al., 2016 [12]	The authors used a distributed cluster-based anchor-free node localization algorithm, where they employed a clustering approach for single-hop nodes and the ToA technique to estimate inter-node distances.
Shah et al., 2020 [21]	The authors proposed an anchor-free algorithm for one-hop nodes by computing the inter-node distances using RSS and the centroid techniques.
Rayavarapu et al., 2021 [17]	The authors applied the LDLA approach and introduced the SFO for implementing range-free anchor-free localization in WSNs using Dv-Hop.
Aka et al., 2023 [13]	The authors grouped nodes into one-hop clusters and estimated distances between nodes within each cluster using RSS.

### 2.2. Propagation Models Relevance

In the context of localization techniques utilizing RSS, it is crucial to consider the precision and variability of the signal at both small and large scales of fading models. Failure to consider these factors can lead to misleading performance assessments on the relevance of any proposed localization solution based on using RSS. Many studies have highlighted the significance of accurate signal propagation models in wireless communication systems, considering phenomena such as multi-path fading and shadowing effects [22].

The lognormal distribution has been traditionally used to represent large-scale shadowing effects [24], but it often appears inflexible due to its relatively inconvenient algebraic representation. In fact, it involves parameters and assumptions that may not always accurately capture the complexity of real-world scenarios, such as variations that can occur in the large-scale shadowing effects in different environments. Therefore, the conditions and assumptions of the lognormal distribution might not hold in practice, leading to a less flexible model, adaptable to changes in environmental conditions or other factors that can influence large-scale shadowing effects. Hence, the gamma distribution has been largely used as a substitute for lognormal distribution [25]. Nevertheless, in a realistic environment, they happen simultaneously; therefore, composite fading distribution is commonly required in wireless communication modeling. However, even if Nakagami-gamma (generalized K) and Nakagami-inverse Gaussian (NiG) approaches model simply the shadowed fading, neither of them endures the match over the whole range of fading and shadowing values [24]. Recent studies and analyses presented in the literature show that the composite Nakagami–lognormal (NL) distribution proposed in [26] to obtain the outage probability has been widely employed to model the mixture of small-scale fading and shadowing, and it fits in more measurement campaigns than the other distributions [27,28].

## 3. Contextual Received Signal Strength Approach

### 3.1. Assumptions

In developing the CRSSA for WSNs, our methodology is predicated on a set of foundational assumptions. These assumptions are designed to mirror realistic deployment scenarios and signal propagation behavior in WSN environments, ensuring that our approach is both practically relevant and scientifically rigorous. Key among these assumptions are:

i. Random 2D Deployment with Unknown Initial Positions: All sensors, unaware of their own positions initially, are randomly deployed across a two-dimensional plane, mirroring diverse real-world deployment scenarios. After deployment, they are assumed to remain stationary. This static nature simplifies the analysis by eliminating the variable of sensor movement, focusing our study on the efficacy of localization under stable conditions.
ii. Adoption of Nakagami–Lognormal Model for Real-World Scenarios: The adoption of the composite Nakagami–lognormal (NL) distribution model is pivotal in accurately representing the signal fading and shadowing effects in various environments. By utilizing this model, the CRSSA effectively adapts to realistic signal propagation scenarios, enhancing the robustness and reliability of the localization process in diverse WSN settings. The composite NL distribution is employed to model the mixture of small-scale fading and shadowing.
iii. Varied and Dynamic Communication Range Based on Propagation Model: While each sensor operates within a communication range *R*, this range is influenced by the specific propagation model in use, particularly the composite NL distribution. This approach acknowledges the variability in signal strength and range due to environmental factors, ensuring a more accurate representation of real-world WSN conditions.
iv. Dynamic Communication Ranges: Given the reliance on the composite NL propagation model, the communication ranges between sensors are dynamic, reflecting the variability in signal propagation. This dynamic range adds realism to our model, acknowledging the fluctuating nature of signal strength in different environmental conditions.
v. Ensuring Comprehensive Network Connectivity: In our model, we assume that the network is fully connected without any disconnected sub-networks, which is crucial for the effective functioning of the CRSSA method. This complete connectivity is essential for preventing information isolation and ensuring that data can flow seamlessly throughout the entire network. To ascertain and maintain this level of network integrity, we utilize Dijkstra’s theorem in our simulations. This helps in identifying the most efficient paths and avoiding scenarios where parts of the network become isolated, thereby safeguarding the reliability and effectiveness of the localization process across the entire network.

These assumptions form the backbone of our approach, allowing us to develop and evaluate the CRSSA in a controlled yet realistic manner, simulating conditions representative of typical WSN applications.

### 3.2. Propagation Model

The communication between two sensors is hence assumed to be affected by both shadowing and small-scale fading resulting from obstructions and multi-path propagation, respectively [28]. In function of the transmitting power, $P_{t}$, the received power at distance *d* is well known [29] and expressed as

(1) $$P_{r}\left(d\right)\left[dBm\right]=P_{t}\left[dBm\right]-PL\left(d\right)\left[dB\right].$$

Antenna gains are included in PL(d), the path loss at distance *d*, defined as follows:

(2) $$PL\left(d\right)=PL\left(d_{0}\right)+10\gamma \left(\frac{d}{d_{0}}\right)+X,$$

where $PL(d_{0})$ is the free space path loss calculated at $d_{0}$, the close-in reference distance, γ is the path loss exponent, (2≤γ≤6), and *X* is the composite when fading and shadowing affect the signal simultaneously, known as Nakagami–lognormal fading. Using the properties and theories in [30,31,32], the pdf of the Nakagami–lognormal (NLN) power *u* is defined by Equation (4.76) in [24], as

(3) $$f_{NLN}\left(u\right)=\int_{0}^{\infty }\frac{m^{m}u^{m-1}}{z^{m}\Gamma \left(m\right)}exp\left(-m\frac{u}{z}\right)\frac{\left(10/log_{e}\left(10\right)\right)}{\sqrt{(}2\pi \sigma^{2}z^{2})}exp[\frac{{\left(10log_{10}z-\mu \right)}^{2}}{2\sigma^{2}}]dz,$$

where *m* is the shape parameter (m≥1/2), Γ(m) is the gamma function, *z* is the average power, and f(z) is treated to be lognormal with mean μ and variance $\sigma^{2}$.

### 3.3. Network Model and Overview

**Figure 1:**
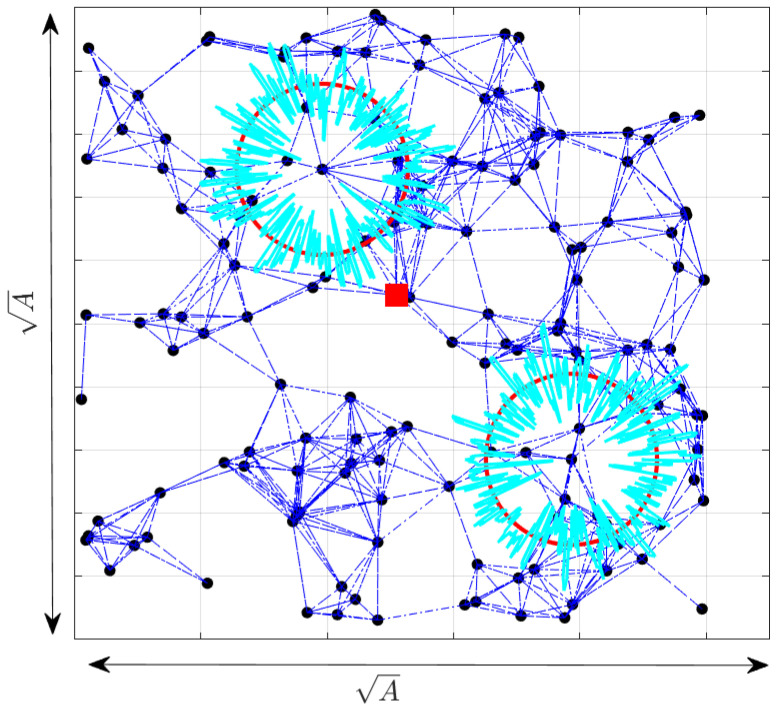
Network Model.

*N* nodes are randomly uniformly deployed in a 2D square area *A* such that $A>>\pi R^{2}$, where *R* is the communication range of each node. We assume a fully connected network, i.e., no node is isolated. Figure 1 illustrates an example of a sensor network where *N* nodes are randomly deployed in a 2D square area. The regular nodes, depicted as blue dots, and a sink node depicted as red square. Unknown nodes are interconnected by dashed lines representing direct or ’one-hop’ connections between two neighboring nodes at a particular instant, underscoring the localized structure of the network at that precise moment. This figure challenges traditional models that typically portray coverage areas as well-defined circles with a fixed radius. Instead, it adopts a more realistic approach by acknowledging the variability and irregularities in signal propagation pertinent to sensor networks. This approach more accurately captures the real-world environmental and spatial challenges faced by sensor networks. As a result, the figure not only depicts standard coverage areas but also those under disturbance, shown in cyan, illustrating the complexities and nuances of data transmission and signal propagation in specific sensor network scenarios.

#### Connectivity

Two nodes (*i* and *j*) are neighbors at one hop if they are connected; hence, $C_{ij}$ is a random variable presenting the connectivity context information defined as

(4) $$C_{ij}=\left\{\begin{array}{cc} 1, & \mathrm{if}\;P_{r}\left(d\right)\geq P_{threshold}\;\\ 0, & \mathrm{otherwise} \end{array}\right.,$$

where i={1,⋯,N}, j={1,⋯,N}, and j≠i. $P_{threshold}$ is the power detection threshold, and *d* is the distance separating *i* and *j*. The global connectivity context information matrix CI is then defined as

(5) $$\mathrm{CI}={\left[C_{ij}\right]}_{N\times N}.$$

### 3.4. CRSS Approach

#### 3.4.1. Analysis from Spatial Data

A complete spatial randomness (csr) is synonymous with a homogeneous Poisson process in $R^{d}$. (“This process has the property that, conditional on N(A). The number of events in a bounded region $A\subset R^{d}$, the events of the process are independently and uniformly distributed over *A*. That is, given N(A)=n, the ordered *n* tuple of events $\left(s_{1},\ldots ,s_{n}\right)$ in $A^{n}$ satisfies $Prob\left(s_{1}\in B_{1},\ldots ,s_{n}\in B_{n}\right)=\prod_{i=1}^{n}\left(\right|B_{i}|/|A|),B_{1},\ldots ,B_{n}\subset A$“) [33] (p. 586). Distances may be measured between sample and nearest events. For a completely spatial process, i.e., a homogeneous Poisson process, the distribution theory for nearest neighbor distances is well known [34], where the density of the positive random variable *W* in $R^{2}$ is

(6) $$g\left(W\right)=2\pi \lambda we^{\left(-\pi \lambda w^{2}\right)}.$$

Theoretical calculations show that, under csr in $R^{2}$, the probability G(r) that the distance from a chosen event to its nearest event is less than or equal to *r* can be expressed by

(7) $$G\left(r\right)=1-e^{\left(-\lambda \pi r^{2}\right)},$$

where λ is the intensity of the homogeneous point process. For the homogeneous Poisson process, the probability that there are no events within distance *x* for an arbitrary point is $e^{\left(-\lambda \pi x^{2}\right)}$. Hence, the distribution function of the point to the nearest event distance is $1-e^{\left(-\lambda \pi x^{2}\right)},x>0$.

#### 3.4.2. From Spatial Data to WSN

Applying this analysis from spatial data, in a sensor network, the random point is the random node (sensor) [35]. Thus, the pdf of the distance, β>0, of a node to its nearest neighbor is

(8) $$f\left(\beta \right)=2\pi \lambda \beta e^{\left(-\lambda \pi \beta^{2}\right)}.$$

Moreover, the probability that this distance is less than or equal to the communication range R is then expressed as

(9) $$P\left(\beta \leq R\right)=\int_{\beta =0}^{R}f\left(\beta \right)d\beta =1-e^{\left(-\lambda \pi R^{2}\right)}.$$

(i) In a one-dimensional case
  *N* nodes are randomly uniformly deployed on a distance $d_{max}=\left[0,x_{max}\right]$. Let *v* be the number of nodes located in an interval $\left[x_{1},x_{2}\right]$ with a probability $p=\frac{x_{2}-x_{1}}{d_{max}}$ [35]. Let ξ be a random variable denoting the number of nodes in a defined interval. Therefore, the probability that *v* of *N* are placed with $\left[x_{1},x_{2}\right]$ is
  (10) P(ξ=v)=Nvpv(1−p)N−v,
  for N>>1 and $\frac{\left(x_{2}-x_{1}\right)}{d_{max}}<<1$, this solution can be approximated with a Poisson distribution, with $p=\frac{x_{2}-x_{1}}{d_{max}}$, and keeping the density $\lambda =\frac{N}{d_{max}}$ constant:
  (11) $$\begin{array}{cc} P(\xi =v) & =\frac{{\left(Np\right)}^{v}}{v!}e^{-Np}\\ \; & =(\frac{N\frac{\left(x_{2}-x_{1}\right)}{d_{max}}}{v!}{)}^{v}e^{-N\frac{\left(x_{2}-x_{1}\right)}{d_{max}}}\\ \; & ={\left(\frac{\lambda (x_{2}-x_{1})}{v!}\right)}^{v}e^{-\lambda (x_{2}-x_{1})} \end{array}$$
  Thus, the probability that a node has *v* neighbors within its communication range *R*, is the same as the probability in Equation (11), but the distance interval is on 2R such as
  (12) $$P\left(\xi =v\right)=(\frac{\lambda 2R}{v!}{)}^{v}e^{-\lambda 2R}.$$
(ii) In two-dimensional deployment

*N* nodes are deployed randomly on surface *A*, providing a realistic representation of random node placement in practical scenarios. Under this assumption, the average number of nodes per unit area is denoted by λ=N/A. This allows us to compute the probability P(ξ=v) that any subset of *v* nodes from the total *N* nodes falls within a specific sub-area $A_{0}$ of the system plane *A*, delimited by the communication range *R* of a node, with $A_{0}=\pi R^{2}$; hence, analogously to (11), P(ξ=v) is obtained as

(13) $$\begin{array}{cc} P(\xi =v) & =\frac{{\left(\frac{A_{0}}{A}N\right)}^{v}}{v!}e^{-\frac{A_{0}}{A}N}\\ \; & =(\frac{\lambda A_{0}}{v!}{)}^{v}e^{-\lambda A_{0}}\\ \; & =(\frac{\lambda \pi R^{2}}{v!}{)}^{v}e^{-\lambda \pi R^{2}}. \end{array}$$

#### 3.4.3. Probabilistic Approach in Interpreting RSS

In our examination of the network’s connectivity, we consider $\bar{R}$ to represent the average effective communication radius, assuming a homogeneous propagation environment. This radius is crucial for estimating the area $\bar{A}$ within which a typical sensor node can communicate, calculated as the area of a circle, $\bar{A}=\pi {\bar{R}}^{2}$. This average range is pivotal in our probabilistic approach for interpreting the RSS values. The expected average number of neighbors falling within a radius R around a given node is described as

(14) $$E\left(\xi \right)=\lambda \pi R^{2}.$$

Acknowledging $\bar{R}$ as an average range allows us to probabilistically interpret the RSS data from sensors, providing a basis for inferring the likelihood of node connectivity within this range. This average range, and the area that it defines, become key in localizing nodes and optimizing the performance of the CRSSA method in a WSN setting to find the number of adjacent or one-hop neighbors for each node, which is pivotal for deducing its communication range.

This calculation represents the expected count of adjacent neighbors for any given node *i*, providing an assessment of the network’s connectivity. On the other hand, the number of connected neighbors, $n_{i}$, to node i=1,⋯,N, can be obtained from the connectivity context data as

(15) $$n_{i}=\sum_{i=1}^{N}C_{ij}.$$

Further, the average number of adjacent neighbors per node is derived by the connectivity matrix considering the individual communication ranges, which may vary due to environmental dynamics but are averaged out across the network:

(16) $$\bar{n}=\frac{1}{N}\;\sum_{i=1}^{N}n_{i}=\;\frac{1}{N}\;\sum_{i=1}^{N}\;\sum_{j=1}^{N}C_{ij}.$$

By leveraging the connectivity matrix, we can accurately determine the average number of connected neighbors for each node, thereby enabling a detailed analysis of network density and communication coverage. This level of specificity is crucial for the effective implementation of the CRSSA method as it directly impacts the localization accuracy within the WSN.

- Range Estimation, $\hat{\bar{R}}$
  The values in Equations (14) and (16) are equivalent; hence, based on these equations, the average range $\bar{R}$ is estimated as
  (17) $$\hat{\bar{R}}=\sum_{i=1}^{N}\frac{\hat{R_{i}}}{N}=\sqrt{\frac{\sum_{i=1}^{N}\sum_{j=1}^{N}C_{ij}}{\lambda \pi N}}.$$

- PLE Estimation
  Theoretical and measured-based propagation models indicate that average received signal power decreases logarithmically with distance [29]. Hence, based on the log-distance path loss model, and assuming the estimated average communication range obtained in Equation (17) as the maximal distance reached by a transmitted signal when the received power measured at this node is equal to the power detection threshold $P_{threshold}$, the PLE, $\hat{\bar{\gamma }}$, can be estimated as
  (18) $$\hat{\bar{\gamma }}=\frac{-P_{threshold}+PL\left(d_{0}\right)}{10log_{10}\left(\hat{\bar{R}}\right)}.$$

- Inter-node distance estimation
  Our proposed approach is an anchor-free solution; thus, it estimates the relative positions with respect to a coordinate system established by a reference group of nodes. Relative positioning information is sufficient for some applications, such as location-aided routing or direction-based routing algorithm [36]. Moreover, the recovery of the network’s key propagation characteristics and its geometry can be addressed by relative positioning mainly from the connectivity information.
  In relative localization, nodes are localized with respect to each other; thus, we estimate the distance separating them. In this work, we will focus on estimating the distance separating two neighbors nodes at one hop. Since the objective is to prove the efficiency of the proposed CRSSA, distances between nodes at more than one hop are beyond the scope of this work and will be addressed in future publications.
  Based on the log-distance path loss model, the value of the estimated PLE in Equation (18), and the received power value obtained at neighbor node *k*, $Pr_{ik}$ is the received power at node *k*, and each node i=1,⋯,N, estimates its distances separating its connected neighbor nodes, $k\neq i\in \kappa =\{i_{1},\cdots ,{\bar{n}}_{i}\}$, as
  (19) $${\hat{d}}_{ik}=10^{\frac{PL\left(d_{0}\right)-Pr_{d_{ik}}}{10\hat{\bar{\gamma }}}}.$$
- Localization of nodes
  To estimate the positions of nodes, we exploit estimated inter-node distances, RSS values, and the sink position. Our approach employs a gradient-based optimization method with weighted residuals, utilizing the RSS values as a key factor in the objective function. Subsequently, we assess the accuracy of the estimated positions through the calculation of the NRMSE.

### 3.5. Flowchart and Algorithm

**Figure 2:**
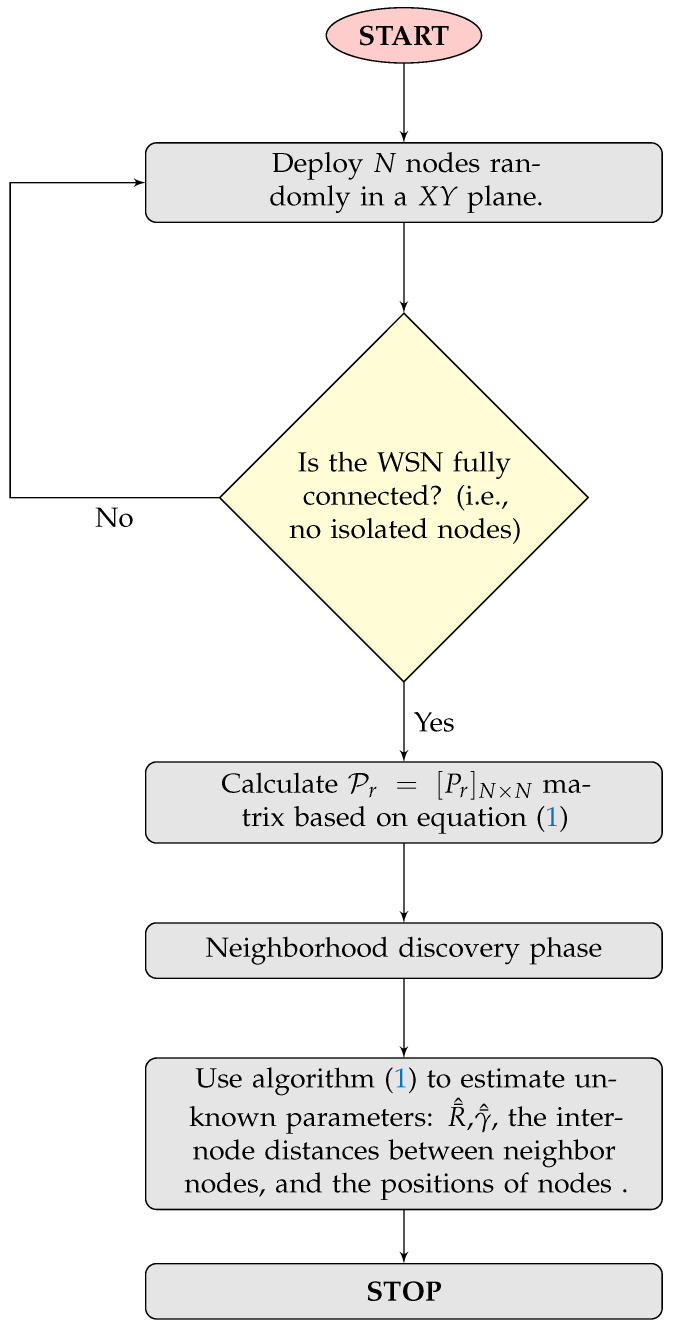
CRSSA flowchart.

To summarize the previous discussion, the flowchart in Figure 2, and the neighbor discovery phase, as well as Algorithm 1, to jointly estimate the PLE value, the inter-node distances are presented below.
**Algorithm 1:** Contextual Received Signal Strength Approach**Input:** *N*: Unknown nodes, **λ**: Network density, *A*: Deployment area**Output:** **$\hat{\bar{R}}$**: Estimated mean range, **$\hat{\bar{\gamma }}$**: Estimated mean path loss exponent, **${\hat{d}}_{ik}$**: Estimated inter-node distance, node position ($\hat{x_{i}},\hat{y_{i}}$)**Data:**  $P_{r}\left(d\right)$: Received power at distance *d***1** **for** *i* = 1 *to* *N* **do**
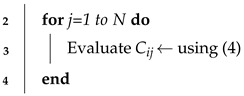
**5** **end****6** **for** *i* = 1 *to* *N* **do**
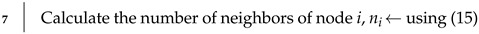
**8** **end****9** Derive the average number of adjacent neighbors $\bar{n}$← using (16)**10** Estimate the mean range $\hat{\bar{R}}$ using (17) **for** *i* = 1 *to* *N* **do**
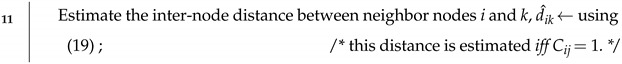
**12** **end****13** Estimate the positions of nodes $\left(\hat{x_{i}},\hat{y_{i}}\right)$ using gradient-based optimization method with weighted residuals.

#### Neighbor Discovery Phase

To build the neighbor connectivity context matrix, each node i(i=1,⋯,N) has to discover its neighbors by sending through the network a HELLO message containing its identity, a single-bit variable *receivedhello* equal to 1 if a full HELLO has been received from the neighbor, and a neighbor Set (*NeighS*), which is updated when a HELLO packet is received from the neighbor. The neighbor connectivity context matrix $C_{ij}$ assigns a value of 0 or 1 for each pair of nodes depending on the neighborhood information. We assume a non-symmetric connectivity context matrix. The value of the matrix is set as follows for each pair of sensors *i* and *j*, where (i=1,⋯,N), j=(1,⋯,N) and j≠i.

1. If *receivedhello* is 1 for both sensors *i* and *j*, $C_{ij}=C_{ji}=1$.
2. If *receivedhello* is 1 for sensor *i* and 0 for sensor *j*, $C_{ij}=1$ and $C_{ji}=0$.
3. If *receivedhello* is 0 for both sensors *i* and *j*, $C_{ij}=C_{ji}=0$.

Once the packet is successfully received by a node, the *NeighS* is updated by adding the identity of the node transmitting the HELLO packet. At this step, each node is aware of its neighbors and their numbers. The information concerning each node connectivity context matrix is sent to the sink node in charge of computational operations. After a successful exchange of HELLO packets, nodes can automatically establish adjacency and can start to send and route data between them. After the neighbor discovery phase, the information on global connectivity is used by the sink node to estimate the channel parameters and the inter-node distances.

## 4. Performance Evaluation

### 4.1. Evaluation Metric

In order to prove the effectiveness of our proposed approach, we calculate the normalized root mean square error (NRMSE) of the estimated inter-node distances as

(20) $$e=\frac{1}{N}\sum_{i=1}^{N}\frac{1}{C(\kappa_{i})}\frac{\sum_{k\in \kappa_{i}}\sqrt{{\left({\hat{d}}_{ik}-d_{ik}\right)}^{2}}}{\bar{R}},$$

where ${\hat{d}}_{ik}$ is the estimated inter-node distance, $d_{ik}$ is its real value, and $C(\kappa_{i})$ is the cardinal of $\kappa_{i}$, the set of node-*i*’s neighbors. The normalization is performed with respect to the the average communication range $\bar{R}$. Other normalization metrics go far beyond the scope of this work. Also, we assess the localization NRMSE as

(21) $$e_{\mathrm{position}}=\frac{1}{N_{u}}\sum_{i=1}^{N_{u}}\frac{\sqrt{{\left(x_{i}-\hat{x_{i}}\right)}^{2}+{\left(y_{i}-\hat{y_{i}}\right)}^{2}}}{\bar{R}},$$

where $N_{u}$ is the number of localized sensors.

### 4.2. Simulation Setup

Extensive simulations for evaluating the performance of our CRSSA are conducted by using Matlab (R2022b) [37]. In order to prove the stability and the efficiency of our approach, we test it in different propagation parameters, as well as different numbers of nodes. The general simulation setup is listed in Table 2.

A random deployment of *N* static nodes inside a square region of length $\sqrt{A}$ in each run is considered. We execute 100 runs, and we make sure that the network is fully connected at each run. Random received power values for each link are generated, following relations explained in Section 3.2. Parameters’ values used for simulation such as γ, σ, and *m* are listed in each subsection below. The path loss exponent, the communication range, and the inter-node distances are then estimated using the generated noisy received power. The CRSS approach is studied by fixing two variables and varying the third one.

In order to test the impact of varying the Nakagami parameter, γ and σ values are fixed and *m* is changed. At each variation of the *m* value, the approach is tested for 100 runs, and the results are reported in Section 4.2.1. Similarly, to assess the impact of the PLE value on the accuracy and the reliability of the CRSS approach, σ and *m* values are fixed, and the γ value is changed; Section 4.2.2 details obtained results. Analogously, Section 4.2.3 elucidates the consequence of varying the shadowing standard deviation on the CRSS approach.

#### 4.2.1. m-Nakagami Parameter Variable

**Figure 3:**
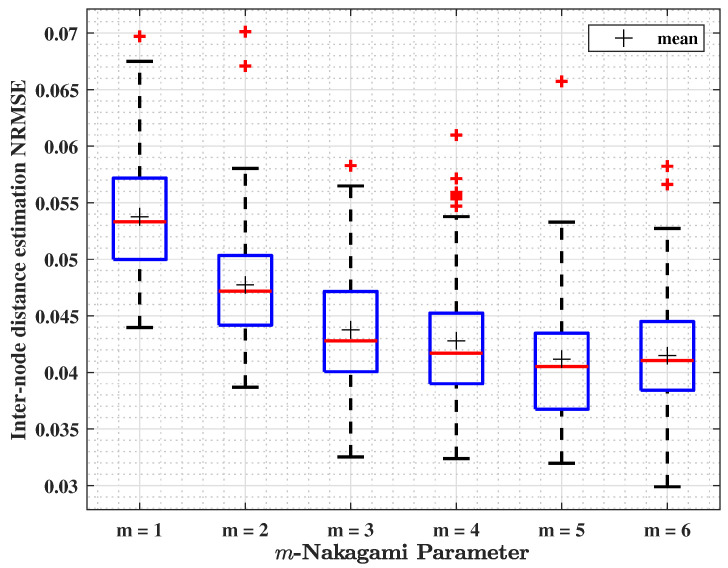
Inter-node distance estimation NRMSE for variable m-Nakagami parameter.

The results presented in Figure 3 show the inter-node distance estimation NRMSE by varying the value of the *m*-Nakagami parameter while γ=3 and σ=4dB. NRMSE is calculated based on Equation (20). The blue boxplots show the results of 100 runs conducted using the parameters listed in Table 3. They show how the data error is extended, and red lines show the median of the error. A significant relationship is identified, as shown in Figure 3, between the Nakagami shape parameter *m* and the localization error. Notably, the results showed that as *m* increases from 1 to 5, the median localization error progressively decreases from 5.5% to 4% of the range. This improvement aligns with the theory that a higher *m* indicates a signal less prone to fading, resulting in greater accuracy. However, a key observation is that the most significant changes occur at m=1, where the model exhibits the most multipath fading. Beyond m=5, improvements become marginal, with the error slightly increasing to 4.01% for m=6. This indicates a plateau in performance gains, influenced by other limiting factors such as the path loss exponent or shadowing. Additionally, it is important to note that, for m=1, the error increases by 1.5% from its ideal level observed at m=5.

#### 4.2.2. γ: PLE Parameter Variable

**Figure 4:**
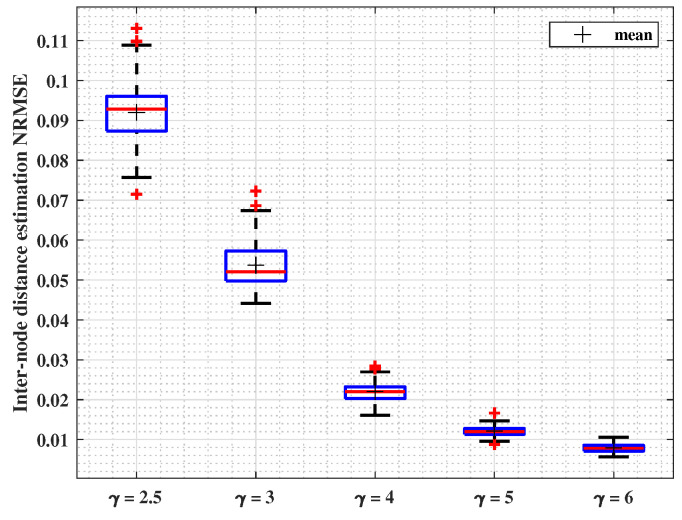
Inter-node distance estimation NRMSE for variable PLE values.

In this subsection, the results presented in Figure 4 show the estimation NRMSE by varying the value of γ while m=4 and σ=4dB. Similarly, boxplots show the results of 100 runs conducted using the parameters listed in Table 4. They show how the data error is extended, and red lines show the median of the error. Low errors show the approach stability. The interval of NRMSE values ranges between 0.01 and 0.11.

Another relationship is identified in this case between the PLE value γ and the localization error. Remarkably, the results showed that as γ increases from 2.5 to 6, the median localization error progressively decreases from 9% to 1% of the range. This enhancement is explained by the fact that smaller PLE values translate into higher average-range values. Thus, the number of neighbors within range, yet at more than a single-hop distance, gets increasingly larger, thus biasing more seriously the estimate in (17).

#### 4.2.3. σ: Lognormal Shadowing Standard Deviation Variable

**Figure 5:**
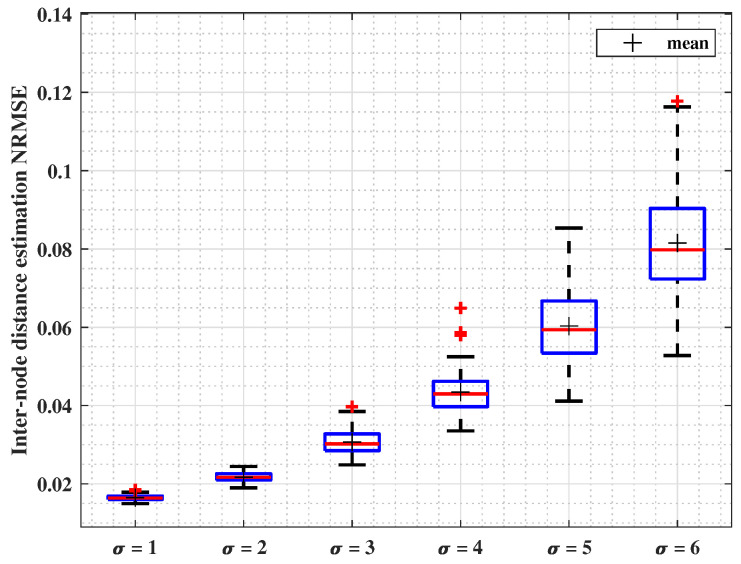
Inter-node distance estimation NRMSE for variable σ, lognormal shadowing standard deviation.

In this subsection, the impact of varying σ is studied. Simulations parameters are listed in Table 5. Figure 5 presents boxplots illustrating the results of 100 runs. They show how the data error is extended, and red lines show the median of the error. It can be observed that NRMSE values increase with the σ value, without exceeding 0.12 when σ=6 dB. Correspondingly to intuitive expectations, where σ introduces randomness in the received signal power, the median localization error progressively increases from 2% to 8% of the range.

#### 4.2.4. Node’s Number Impact

This approach relies on the network contextual information. The node’s number impact study is crucial as it affects the network density, λ. In fact, this latter is a key parameter studied in different WSN applications. Bulusu [38] proposed Equation (22) to calculate λ as

(22) $$\lambda =\frac{N\pi R^{2}}{A},$$

where *N* is the number of nodes in area *A*, and *R* is the nominal range of each node. However, the authors in [39] propose a more precise equation as

(23) $$\lambda =\;lim_{|A|\to 0}\frac{N(A)}{\left|A\right|}$$

where density is measured in nodes per $m^{2}$.

A higher node density introduces less distance between nodes; thus, the impact of this network parameter is crucial in this study since it affects the number of neighbor nodes within a communication range.

**Figure 6:**
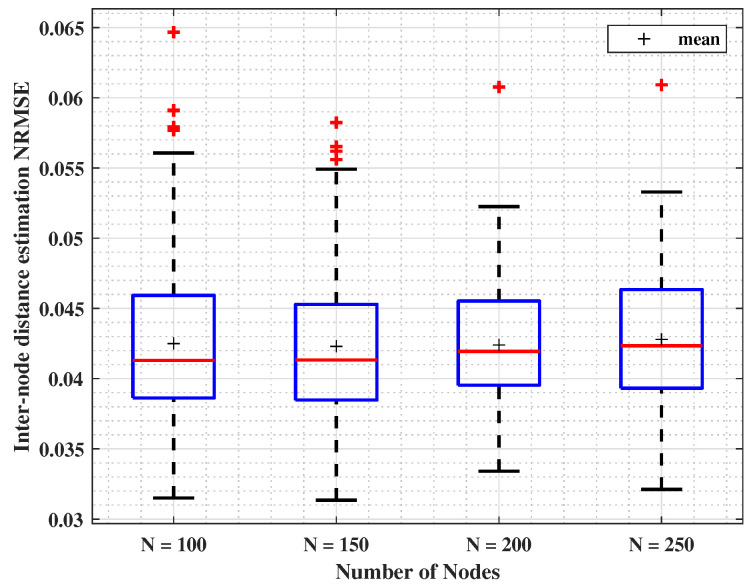
Inter-node distance estimation NRMSE for different number of nodes, *N*, with (γ=3,σ=4, m=4).

The CRSSA is tested with a different number of nodes without changing the deployment area. Boxplots in Figure 6 show the simulation results for different *N*, by illustrating how errors are extended. Simulation parameters are listed in Table 3. As observed, the maximal NRMSE value does not exceed 0.065 for N=100. An important observation is drawn from Figure 6: the median localization error denoted by red lines remains stable when varying *N*, where it changes from 4.1% to 4.2% of the range. Once more, the results indicate a plateau in performance gains and validate the effectiveness of the approach.

#### 4.2.5. Results Comparison

After proving the competence of the CRSSA in different and varying channel parameters and numbers of nodes, we evaluate its performance by comparing the NRMSE against state-of-the-art anchor-free localization techniques proposed in recent studies, namely, Shah et al. [21], SFO [17], and EAFLA [13]. Shah et al. employed a time division multiple access (TDMA) scheduling algorithm, where each node broadcasts a beacon message containing its unique node ID. RSS measurements are then utilized to estimate distances between nodes, and the centroid and average distance are computed for localization.

The SFO technique applied in [17] involves an approach using the DV-Hop algorithm. This technique leverages the principles of SFO to optimize node positions based on hop distances obtained from the DV-Hop algorithm.

The EAFLA proposed in [13] begins by electing cluster heads and forming clusters according to the low-energy adaptive cluster hierarchical (LEACH) protocol. Distances between nodes are then estimated using a combination of the RSS signal transmission model and the Kleinrock and Sylvester model [14].

**Figure 7:**
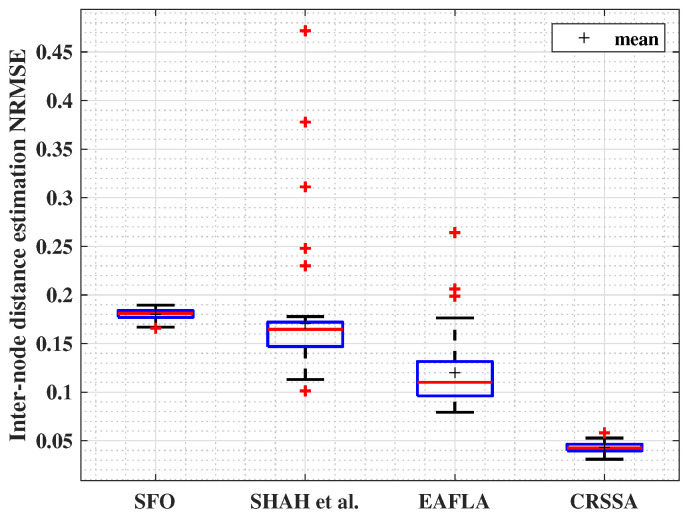
Inter-node estimation NRMSE comparison (γ=3,σ=4, and m=4).

In Figure 7, we evaluate the NRMSE under specific parameter settings (γ=3, σ=4, and m=4). We present results in blue boxplots. Our proposed approach consistently outperforms existing benchmark methods found in the literature. The NRMSE achieved by our approach falls within an impressive range of [0.03,0.058]. In comparison, errors obtained using the SFO method vary between 0.16 and 0.189, Shah et al.’s approach yields errors within [0.45,0.1], and errors with the EAFLA range from 0.07 to 0.26. This demonstrates the superior accuracy and precision of our proposed method, consistently achieving lower NRMSE values across the specified parameter configurations when compared to existing state-of-the-art approaches.

#### 4.2.6. Node Localization

**Figure 8:**
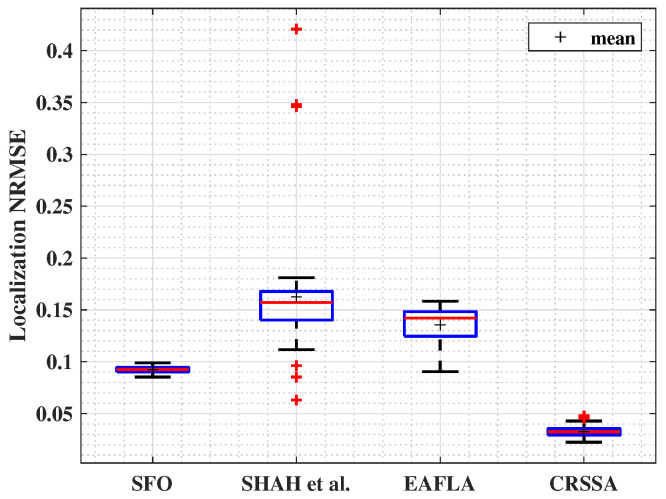
Localization NRMSE (γ=3,σ=4, and m=4).

In order to localize sensors, we adopt a gradient-based optimization method with weighted residuals, utilizing the RSS values as a key factor in the objective function. Neighbors at one hop of each node are positioned using the estimated inter-node distances. The sink’s neighbors are localized based on the known position of the sink, and then the rest of the sensors are positioned. Figure 8 shows that our proposed methodology consistently surpasses other anchor-free localization techniques found in the literature. The NRMSE achieved by our approach falls within a range of [0.02, 0.045]. In contrast, errors obtained using the SFO method vary between 0.089 and 0.16, Shah et al.’s approach yields errors within [0.12, 0.18], and errors with the EAFLA range from 0.09 to 0.16. This highlights the superior accuracy and precision of our proposed method, consistently attaining lower NRMSE values.

#### 4.2.7. Discussion

As presented above, we aim through extensive simulations to show the efficiency of our approach by achieving low errors in estimating inter-node distances in a WSN, as well as its stability when varying different propagation parameters in conditions simulating a realistic model. Moreover, our technique outperforms the benchmark anchor-free approach in the same propagation models and same assumptions.

Hence, our anchor-free technique relies on using the contextual network information consisting of connectivity, network density, and node number to overcome any variation in the WSN deployment environment. In other words, our technique adjusts and adapts the estimation of the main unknown parameters, $\hat{\bar{R}}$ and $\hat{\bar{\gamma }}$, by using derived equation functions of propagation model parameters as well as deployed network parameters. After that, $\hat{\bar{R}}$ and $\hat{\bar{\gamma }}$ are used in estimating the inter-node distance based on the received power model, allowing us to accurately find the relation between distance and received power without using any extra device or known nodes. Then, the nodes are localized based on the estimated inter-node distances. This will drastically decrease the cost of the WSN since no anchor nodes are needed and the aim is to achieve a relative positioning in some WSN applications.

## 5. Conclusions and Future Work

In this paper, we present an anchor-free localization technique in 2D WSN. Analytical expressions for the average communication range, PLE value, and inter-node distances are derived. The introduction of the CRSSA in this article marks a substantial advancement in wireless sensor networks (WSNs), showcasing a novel anchor-free localization technique that leverages RSS and contextual connectivity. This approach sets a new standard in localization accuracy within WSNs, highlighting the study’s innovative edge and technical proficiency. The study’s robust theoretical framework and comprehensive simulative evaluations are particularly noteworthy, demonstrating the method’s effectiveness and superiority in precision compared to existing localization methods like EAFLA. Additionally, the anchor-free nature of the CRSSA emerges as a pivotal development, offering enhanced flexibility and cost-effectiveness in sensor network deployments, which are crucial in the evolving realm of wireless communications.

The potential of the CRSSA in the field of WSNs is underscored by its adaptability and economic efficiency. Future research avenues, such as addressing real-time computational efficiency and undertaking experimental validations in diverse real-world settings, will further cement its practicality and application scope. Integrating the CRSSA with emerging technologies and considering dynamic environments where node mobility is a factor will expand its utility and relevance.

This study, therefore, not only provides a significant contribution to WSN localization techniques but also opens up exciting possibilities for future innovation and practical applications, setting a new benchmark in the field. 

## Figures and Tables

**Table 2:** WSN simulation parameter setup.

Parameters [Unit]	Values (s)
$\sqrt{A}\left[m\right]$: Square length	100
*N*: Sensor node cardinality	150
$P_{threshold}$: Threshold power [dBm]	−90
$PL(d_{0})$: Free space path loss at $d_{0}$ [dB]	−45
$P_{t}$: Transmitting power [dBm]	0
η: Number of runs	100

**Table 3:** Variable parameter setup 1.

Parameters [Unit]	Values (s)
γ: PLE	3
σ: Lognormal shadowing standard deviation [dB]	4
*m*: Nakagami parameter	(1;2;3;4;5;6)

**Table 4:** Variable parameter setup 2.

Parameters [Unit]	Values (s)
γ: PLE	(2.5;3;4;5;6)
σ: Lognormal shadowing standard deviation [dB]	4
*m*: Nakagami parameter	4

**Table 5:** Variable parameter setup 3.

Parameters [Unit]	Values (s)
γ: PLE	3
σ: Lognormal shadowing standard deviation [dB]	(1;2;3;4;5;6)
*m*: Nakagami parameter	4

## Data Availability

Data is contained within the article.

## Abbreviations

The following abbreviations are used in this manuscript:

RSS	Received Signal Strength
WSNs	Wireless Sensor Networks
PLE	Path Loss Exponent
CRSS	Contextual Received Signal Strength
NRMSE	Normalized Root Mean Square Error
